# Survival Impact of Programmed Cell Death Ligand‐1 Expression in Patients With Epithelial Ovarian Carcinoma

**DOI:** 10.1002/cnr2.70636

**Published:** 2026-07-30

**Authors:** Achjima Tankul, Suchanan Hanamornroongruang, Naravat Poungvarin, Rachawalan Suriyasaengsri, Sompop Kuljarusnont, Pornprom Ittiamornlert, Wathirada Karnchanabanyong, Irene Ruengkhachorn, Saowalak Hunnangkul

**Affiliations:** ^1^ Gynecologic Oncology Division, Department of Obstetrics & Gynecology, Faculty of Medicine Siriraj Hospital Mahidol University Bangkok Thailand; ^2^ Charoenkrung Pracharak Hospital Bangkok Thailand; ^3^ Department of Pathology, Faculty of Medicine Siriraj Hospital Mahidol University Bangkok Thailand; ^4^ Department of Clinical Pathology, Faculty of Medicine Siriraj Hospital Mahidol University Bangkok Thailand; ^5^ Division of Forensic Science Standard Central Institute of Forensic Science Bangkok Thailand; ^6^ Division of Clinical Epidemiology, Department of Research and Development, Faculty of Medicine Siriraj Hospital Mahidol University Bangkok Thailand

**Keywords:** biomarkers, cancer, ovary, PD‐L1, survival

## Abstract

**Aims:**

To evaluate the proportion of expressed programmed cell death ligands‐1 (PD‐L1) in epithelial ovarian carcinoma patients, and the association with the clinicopathological, surgical outcomes, and oncologic outcomes. To compare PD‐L1 protein expression with messenger ribonucleic acid (mRNA) expression.

**Methods:**

The patients who had pathology of epithelial ovarian/tubal/peritoneal carcinoma were enrolled. The demographic data, tumor characteristics, surgical outcomes, treatment details, and treatment response were analyzed by descriptive statistics. Spearman's rank correlation coefficient test was used to determine the correlation between protein expression and mRNA expression. The survival curve was developed utilizing the Kaplan‐Meier method, the log‐rank test was used to compare survival distributions of patients according to the PD‐L1 protein expression.

**Results:**

A total of 100 patients, with high‐grade serous carcinoma being the most common subtype (42%). Advanced‐stage disease was present in 49% of patients, and 65% achieved optimal cytoreduction. PD‐L1 protein expression was detected in 23% of tumors, while the median normalized mRNA expression was 0.015 (IQR 0.004–0.037). A moderate positive correlation was observed between PD‐L1 protein and mRNA expression (Spearman's rho = 0.407, *p* < 0.001). The mean progression‐free survival (PFS) and overall survival (OS) were 44.32 ± 4.04 months and 74.19 ± 3.33 months, respectively.

**Conclusions:**

PD‐L1 protein expression was observed in a minority of patients and was not significantly associated with survival outcomes. There was a moderate correlation between PD‐L1 protein and mRNA expressions.

## 1 Introduction

Epithelial ovarian carcinoma (EOC) is one of the leading causes of death among gynecological malignancies. Most patients are diagnosed at an advanced stage. Standard treatment consists of cytoreductive surgery followed by platinum‐taxane‐based chemotherapy with or without bevacizumab. Intensification with additional cytotoxic agents has not demonstrated a survival benefit. Despite aggressive treatment, the 5‐year overall survival (OS) remains approximately 30% [1, 2, 3, 4]. Targeted drug combinations with chemotherapy have improved oncological outcomes in both primary and recurrent settings [5, 6, 7, 8, 9]. Genetic mutations and biomarker profiles are increasingly being used. BRCA1/2 mutations are reported to be good predictive biomarkers for sensitivity to poly ADP‐ribose polymerase (PARP) inhibitors [10, 11, 12]. Novel treatment modalities are needed, and a variety of targeted therapies directed against cancer molecular targets and tumor microenvironments are being developed.

Recent advances in immuno‐oncology have demonstrated that the tumor immune microenvironment plays a critical role in cancer progression and therapeutic response. Immune checkpoint pathways, particularly the programmed cell death‐1 (PD‐1)/programmed cell death ligand‐1 (PD‐L1) axis, contribute to immune evasion by inhibiting cytotoxic T‐cell activity. Blockade of this pathway has shown clinical activity in a subset of patients with recurrent EOC, highlighting the biological relevance of PD‐L1 expression in this disease [12, 13, 14, 15]. However, the prognostic significance of PD‐L1 expression in EOC remains controversial. Several studies have reported that high PD‐L1 expression is associated with poor progression‐free survival (PFS) and OS [16, 17], whereas others have suggested a favorable or neutral prognostic effect [18]. These discrepancies may be attributable to heterogeneity in histologic subtypes, ethnic populations, antibody clones, scoring systems, tissue sampling techniques, and the specific cellular compartments evaluated.

Importantly, most published studies have been conducted in Western populations, and data from Southeast Asia remain limited. Given potential ethnic and biological differences in tumor–immune interactions, region‐specific data are essential to clarify the clinical and biological significance of PD‐L1 in EOC. Moreover, although immunohistochemistry is the most widely used method for PD‐L1 assessment, the relationship between PD‐L1 protein expression and messenger RNA (mRNA) levels in ovarian cancer remains poorly characterized, and discordance between transcriptomic and proteomic expression has been reported in other malignancies.

Therefore, we conducted a prospective cohort study in Thai patients with epithelial ovarian, fallopian tube, and primary peritoneal carcinoma to (1) determine the proportion of PD‐L1 expression in tumor cells, (2) evaluate the association of PD‐L1 expression with clinicopathological characteristics, surgical outcomes, and survival, and (3) examine the correlation between PD‐L1 protein and mRNA expression. This study aims to provide biological insight into PD‐L1 regulation in an underrepresented population and to clarify its potential role as a biomarker in EOC.

## 2 Materials and Methods

### 2.1 Study Design, Setting, and Period

This study was designed as a prospective cohort study conducted at Siriraj Hospital, Faculty of Medicine Siriraj Hospital, Mahidol University, Bangkok, Thailand. Patients were enrolled between November 2016 and February 2018, with longitudinal follow‐up for oncologic outcomes obtained through retrospective medical record review and national registry data. The study protocol was approved by the Siriraj Institutional Review Board (COA no. Si 694/2016 for tissue collection and COA no. Si 263/2024 for survival follow‐up) and was registered with the Thai Clinical Trials Registry (TCTR20161115004).

### 2.2 Participants

Eligible patients were ≥ 18 years old who were able to communicate effectively in Thai and were scheduled to undergo an exploratory laparotomy following a provisional diagnosis of epithelial ovarian/fallopian tube/primary peritoneal carcinoma. They were counseled on the study protocol and signed an informed consent before participation. The primary cytoreductive surgery comprised a total abdominal hysterectomy with bilateral salpingo‐oophorectomy, with maximum cytoreductive effort for patients at a clinically advanced stage or complete surgical staging procedures for patients who appeared to be at an early stage.

During the operation, the tissues that were most suspicious of malignancy were sampled by the A.T., ensuring that there was adequate tissue for pathology interpretation. The sampled tissue was preserved in RNA*later* RNA Stabilization Reagent (Qiagen, Hilden, Germany) for mRNA extraction and analysis. After the final pathology was reported, patients who had non‐epithelial ovarian/fallopian tube/primary peritoneal carcinoma were excluded. The tissue was processed for formalin fixation, and the tissue microarray was processed for immunohistochemistry assessment.

### 2.3 Immunohistochemistry

A tissue microarray was prepared. Immunohistochemistry staining to detect.

**FIGURE 1:**
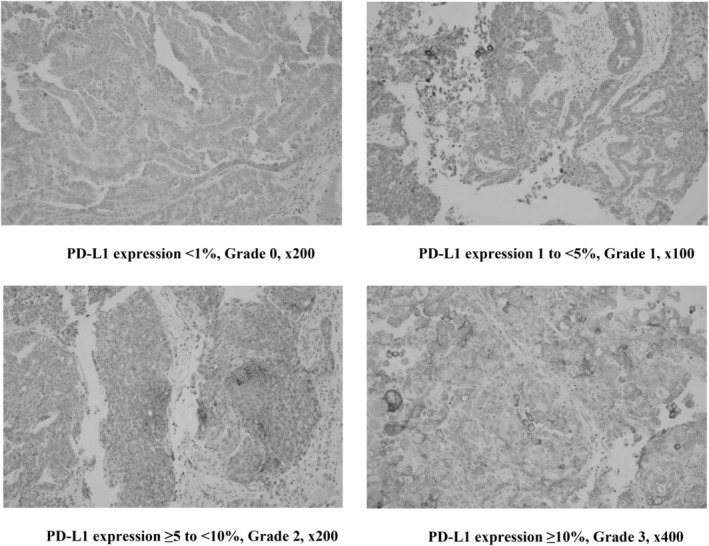
Programmed cell death ligands‐1 expression as detected by immunohistochemistry. Immunohistochemistry staining was classified into 4 grading: < 1% as grade 0 (×200 magnification), 1% to < 5% as grade 1 (×100 magnification), 5% to < 10% as grade 2 (×200 magnification), and ≥ 10% as grade 3 (×400 magnification).

PD‐L1 expression in cancer cells by PD‐L1 immunohistochemistry 28‐8 was performed on a Dako platform. Each slide contained a positive and a negative control. The staining was evaluated by a Gynecologic Pathologist (S.Ha.) according to the manufacturer's protocol. According to the manufacturer's instructions, sample tissues with < 100 tumor cells were not interpretable and were excluded from the study. The expression was classified into 4 grades: < 1% as grade 0, 1 to < 5% as grade 1, 5 to < 10% as grade 2, and ≥ 10% as grade 3 (Figure 1). PD‐L1 positive staining was defined as complete circumferential and/or partial linear plasma membrane staining of cancer cells at any intensity. Cytoplasmic staining, if present, was not considered for scoring purposes.

The semi‐quantitative four‐tier grading system was selected to capture the full dynamic range of tumor cell PD‐L1 expression in this biologically exploratory study. This approach has been used in prior translational studies to evaluate expression heterogeneity beyond dichotomous thresholds. Because this cohort did not include patients treated with immune checkpoint inhibitors and tumor immune‐cell staining was not systematically evaluated, clinically used scoring systems such as the combined positive score (CPS) or tumor proportion score (TPS) were not applied.

All PD‐L1 immunohistochemical slides were independently evaluated by an experienced gynecologic pathologist who was blinded to clinical outcomes and followed the manufacturer's scoring guidelines.

### 2.4 Messenger Ribonucleic Acid Normalized Expression

The total RNA was isolated from 15 mg of tissue using AllPrep DNA/RNA Mini Kit according to the manufacturer's protocol (Qiagen, Hilden, Germany). The complementary deoxyribonucleic acid (cDNA) was synthesized from 200 ng of total RNA by Omniscript RT Kit (Qiagen, Hilden, Germany). Quantitative real‐time PCR (qPCR) was performed in triplicate using previously published primer sequences and PCR conditions with minor modifications [19, 20, 21]. Briefly, a 15‐μL reaction mixture contains of 7.5 μL of QuantiFast SYBR Green Master Mix (Qiagen, Hilden, Germany), 75 nM of primers, and 1 ng of cDNA. Lightcycler 480 system (Roche Diagnostics, Basel, Switzerland) was used to perform qPCR. Expression of eight candidate reference genes, that is, *EEF1A1, EEF1G, RPL13A, B2M, QARS, PPIA, TBP*, and *RPS13* was analyzed in 30 cDNA samples. The two most stable housekeeping genes (HKG) determined by geNorm were Eukaryotic Translation Elongation Factor 1 Alpha 1 (*EEF1A1*) and TATA‐Box Binding Protein (*TBP*) [22]. The quantitative PD‐L1 expression data were normalized to the expression of these genes. The samples with no detectable HKG expression were considered of poor quality.

### 2.5 Definition of Clinical Outcomes

The data gathered included demographic information, tumor characteristics, treatment data, and surgical outcomes. The demographic data comprised age, body mass index (BMI), menopausal status, parity, underlying diseases, and presenting symptoms. BMI was stratified according to the World Health Organization (WHO) recommendations into three groups: < 18.5 kg/m^2^ (underweight), 18.5–24.9 kg/m^2^ (normal BMI), and ≥ 25.0 kg/m^2^ (overweight and obese). The tumor characteristics included gross appearance, histopathologic type, International Federation of Gynecology and Obstetrics (FIGO) stage, and tumor grade. Histopathology was classified according to the WHO guidelines. Surgical outcomes were graded as optimal (residual tumor ≤ 1 cm) or suboptimal (residual tumor > 1 cm). The follow‐up clinical data were retrieved from medical records. For patients who were lost to follow‐up or could not be contacted, survival data were obtained from resident registration units.

### 2.6 Study Endpoints

The primary endpoint of this study was the proportion of PD‐L1 protein expression in tumor cells as determined by immunohistochemistry. Secondary endpoints included (1) the correlation between PD‐L1 protein and mRNA expression, (2) the association of PD‐L1 expression with clinicopathological characteristics and surgical outcomes, and (3) PFS and OS according to PD‐L1 expression status.

### 2.7 Statistical Analysis

SPSS Statistics for Windows, version 29.0 (IBM, Armonk, New York, USA) was used for the statistical analysis. The Kolmogorov–Smirnov test was used to test the normality of continuous data. Demographic data were summarized using descriptive statistics. Data were presented as numbers and percentages, mean ± standard deviation (SD), or median (interquartile range, IQR). Categorical data were presented as numbers and percentages. The categorical variables were compared with either the chi‐square or Fisher's exact test. The normality continuous variables were compared by the independent *t*‐test, and the non‐normality continuous variables were compared by the Mann–Whitney test or Kruskal–Wallis test. In addition to the primary and secondary analyses, exploratory subgroup analyses were performed to evaluate PD‐L1 protein and mRNA expression according to disease stage, histologic subtype, tumor grade, surgical outcome, and treatment response. Spearman's rank correlation test was used to determine the correlation between the PD‐L1 protein and mRNA expressions. The Kaplan–Meier method was used to estimate survival curves, and the log‐rank test was used to compare survival distributions among patients based on PD‐L1 protein expression. Cox proportional hazards regression analysis was performed to evaluate the association between prognostic factors and survival outcomes. The results are presented as hazard ratios (HRs) with 95% confidence intervals (CIs). A *p* value of < 0.05 was considered statistically significant.

## 3 Results

**FIGURE 2:**
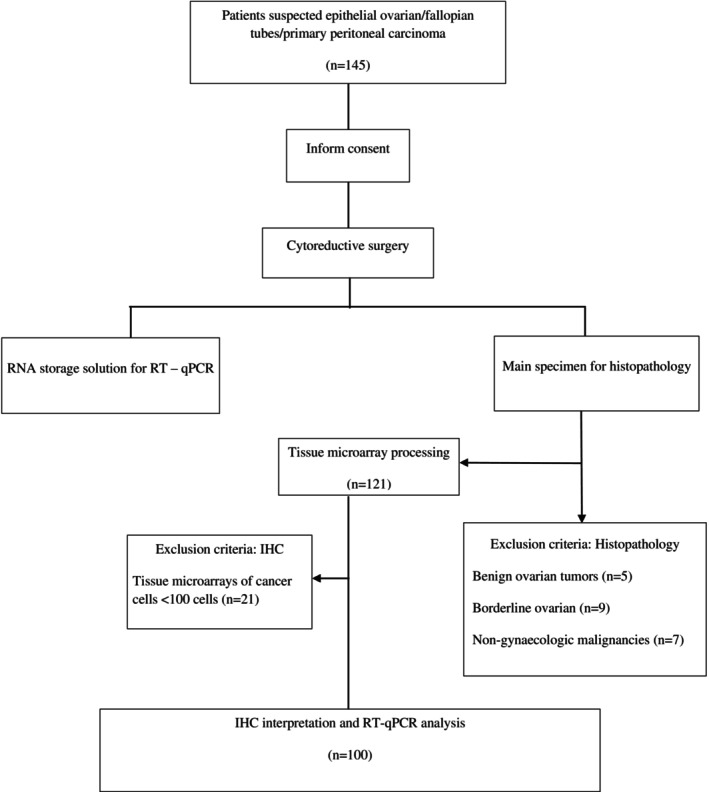
The study procedures and enrolled populations. IHC, immunohistochemistry; *N*, number; RT‐qPCR, quantitative reverse transcription polymerase chain reaction.

A total of 145 patients with epithelial ovarian/fallopian tube/primary peritoneal carcinoma were enrolled between November 2016 and February 2018. Of those, 45 samples were excluded: 24 patients were diagnosed with non‐epithelial ovarian/fallopian tube/primary peritoneal carcinoma, and 21 patients had < 100 cancer cells in their tissue microarray specimens. Thus, a total of 100 patients were entered into the analysis, as shown in Figure 2. The median age was 53.0 years (IQR, 48.3–62.0). The median BMI was 22.5 kg/m^2^ (IQR, 20.3–26.0). Four patients had a history of breast cancer. Two patients had a history of hysterectomy due to myoma uteri. Ninety‐six patients received adjuvant platinum‐based chemotherapy combined with paclitaxel. The mean PFS was 44.32 ± 4.04 months, and the mean OS was 74.19 ± 3.33 months. PD‐L1 protein expression was detected in 23% of patients: nine patients had grade 1 staining, six had grade 2, and eight had grade 3. In exploratory subgroup analyses, the median normalized mRNA expression in all study patients was 0.015 (IQR, 0.004–0.037), and the median mRNA expression relative to the lung cancer cell line (NCI‐H1975) was 0.083 (IQR, 0.024–0.204).

**FIGURE 3:**
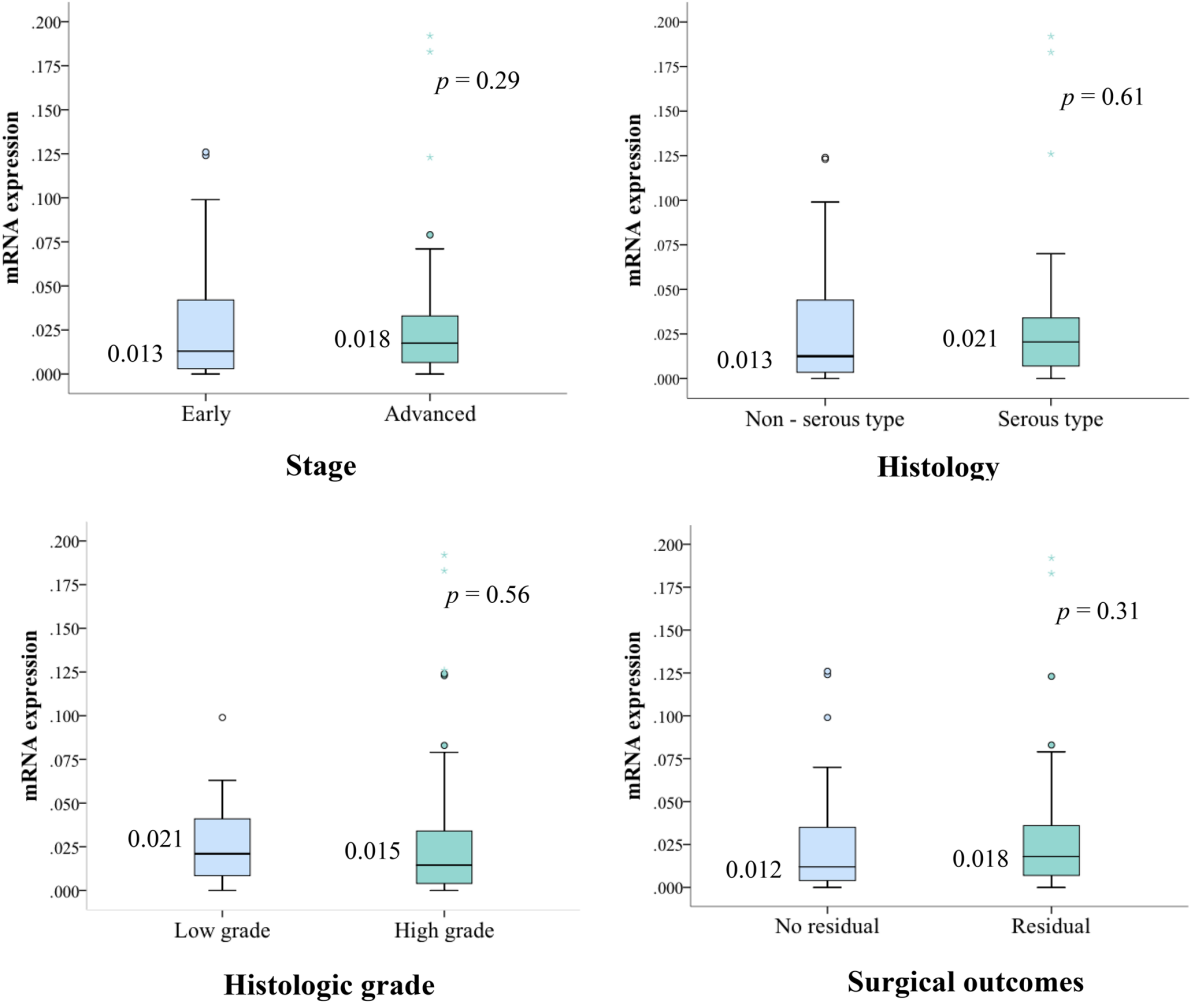
The expression of messenger ribonucleic acid (mRNA) classified as cancer stage, histology, and surgical outcomes. mRNA, messenger ribonucleic acid.

The clinicopathological characteristics of the expression of PD‐L1 by immunohistochemistry were summarized in Table 1. The expression of mRNA classified as cancer stages, histology, and surgical outcomes is shown in Figure 3. The correlations between the clinical characteristics and response assessments and PD‐L1 protein and mRNA expression are presented in Table 2. A moderate positive correlation was observed between PD‐L1 protein and mRNA expression (Spearman's rho = 0.407, *p* < 0.001).

**TABLE 1:** Histopathological characteristic and surgical outcomes with programmed cell death ligands‐1 protein expression in 100 study patients.

Characteristics	Number	Immunohistochemistry of PD‐L1			
		Grade 0 *n* (%)	Grade 1 *n* (%)	Grade 2 *n* (%)	Grade 3 *n* (%)
Age, year					
< 50	27	22 (81.5)	2 (7.4)	2 (7.4)	1 (3.7)
≥ 50	73	54 (75.0)	7 (9.7)	4 (5.6)	7 (9.7)
Body mass index, kg/m^2^					
< 18.5	10	7 (70.0)	1 (10.0)	1 (10.0)	1 (10.0)
18.5–24.9	58	46 (79.3)	4 (6.9)	2 (3.4)	6 (10.3)
≥ 25	32	24 (75.0)	4 (12.5)	3 (9.4)	1 (3.1)
Menopausal status					
Premenopause	34	27 (79.4)	2 (5.9)	2 (5.9)	3 (8.8)
Postmenopause	66	50 (75.8)	7 (10.6)	4 (6.1)	5 (7.6)
Primary sites					
Ovary	93	73 (78.5)	6 (6.5)	6 (6.5)	8 (8.6)
Fallopian tubes	3	1 (33.3)	2 (66.7)	0	0
Peritoneum	7	3 (75.0)	1 (25.0)	0	0
Histopathology					
Serous carcinoma	42	32 (76.2)	5 (11.9)	4 (9.5)	1 (2.4)
Clear cell carcinoma	30	22 (73.3)	1 (3.3)	2 (6.7)	5 (16.7)
Endometrioid carcinoma	16	13 (81.3)	1 (6.3)	0	3 (12.5)
Mucinous carcinoma	4	3 (75.0)	1 (25.0)	0	0
Mixed types	5	5 (100.0)	0	0	0
Carcinosarcoma	2	1 (50.0)	1 (50.0)	0	0
Brenner	1	1 (100.0)	0	0	0
Histology grading					
Well differentiation	11	9 (81.8)	1 (9.1)	0	1 (9.1)
Moderately differentiation	9	7 (77.8)	1 (11.1)	0	1 (11.1)
Poorly differentiation	79	61 (76.3)	7 (8.8)	6 (7.5)	6 (7.5)
Undifferentiation	1	1 (100.0)	0	0	0
FIGO stage					
IA	6	4 (66.7)	0	1 (16.7)	1 (16.7)
IB	0	0	0	0	0
IC					
IC1	15	13 (86.7)	0	0	2 (13.3)
IC2	6	4 (66.7)	1 (16.7)	0	1 (16.7)
IC3	6	4 (66.7)	1 (16.7)	0	1 (16.7)
IIA	5	4 (80.0)	0	1 (20.0)	0
IIB	13	10 (76.9)	1 (7.7)	1 (7.7)	1 (7.7)
IIIA					
IIIA1	2	2 (100.0)	0	0	0
IIIA2	2	1 (50.0)	0	0	1 (50.0)
IIIB	9	6 (66.7)	2 (22.2)	1 (11.1)	0
IIIC	31	24 (77.4)	4 (12.9)	2 (6.5)	1 (3.2)
IVA	3	3 (100.0)	0	0	0
IVB	2	2 (100.0)	0	0	0
Surgical outcomes					
Optimum					
No residual	52	42 (80.8)	2 (3.8)	3 (5.8)	5 (9.6)
Residual ≤ 1 cm	13	8 (61.5)	1 (7.7)	2 (15.4)	2 (15.4)
Suboptimum	35	27 (77.1)	6 (17.1)	1 (2.9)	1 (2.9)
Response assessment					
Complete response	72	54 (75.0)	7 (9.7)	5 (6.9)	6 (8.3)
Partial response	5	5 (100.0)	0	0	0
Stable disease	6	4 (66.7)	1 (16.7)	1 (16.7)	0
Progressive disease	12	11 (91.7)	1 (8.3)	0	0
Loss to assessment	5	3 (60.0)	0	0	2 (40.0)
Recurrence					
Not recurrence	35	29 (82.9)	4 (11.4)	1 (2.9)	1 (2.9)
Had recurrence	60	45 (75.0)	5 (8.3)	5 (8.3)	5 (8.3)
Loss to follow‐up	5	3 (60.0)	0	0	2 (40.0)
Survival					
Alive	62	49 (79.0)	4 (6.5)	3 (4.8)	6 (9.7)
Death	38	28 (73.7)	5 (13.2)	3 (7.9)	2 (5.3)

Abbreviations: FIGO, the International Federation of Gynecology and Obstetrics; PD‐L1, programmed cell death ligands‐1.

**TABLE 2:** The expressions of programmed cell death ligands‐1 protein and messenger ribonucleic acid, classified as clinicopathological and surgical outcomes.

Variables	Protein expression by IHC					Normalized mRNA by RT‐qPCR	
	Grade 0	Grade 1	Grade 2	Grade 3	*p*	Median (IQR)	*p*
	*n* (%)	*n* (%)	*n* (%)	*n* (%)			
Stage groups							
Early	39 (76.5)	3 (5.9)	3 (5.9)	6 (11.2)	0.396	0.013 (0.003–0.043)	0.290
Advanced	38 (77.6)	6 (12.2)	3 (6.1)	2 (4.1)		0.018 (0.006–0.035)	
Histology groups							
Serous	32 (76.2)	5 (11.9)	4 (9.5)	1 (2.4)	0.169	0.021 (0.007–0.034)	0.610
Non serous	45 (77.6)	4 (6.9)	2 (3.4)	7 (12.1)		0.013 (0.003–0.045)	
Histopathology grade							
Low grade	16 (80.0)	2 (10.0)	0	2 (10.0)		0.021 (0.007–0.044)	
High grade	61 (76.3)	7 (8.8)	6 (7.5)	6 (7.5)	0.641	0.015 (0.004–0.035)	0.560
Surgical outcomes							
No residual	42 (80.8)	2 (3.8)	3 (5.8)	5 (9.6)	0.155	0.012 (0.004–0.036)	0.310
Residual ≤ 1 cm	8 (61.5)	1 (7.7)	2 (15.4)	2 (15.4)		0.027 (0.011–0.077)	
Residual > 1 cm	27 (77.1)	6 (17.1)	1 (2.9)	1 (2.9)		0.015 (0.006–0.034)	
Response assessment (*n* = 95)							
Response (CR/PR)	59 (76.6)	7 (9.1)	5 (6.5)	6 (7.8)	0.173	0.015 (0.004–0.035)	0493
Not response	15 (83.3)	2 (11.1)	1 (5.6)	0		0.013 (0.008–0.051)	
PFS (*n* = 95), months					0.734		
Median	19.5	17.9	27.5	13.6	0.425		
< 6 months	12 (85.7)	1 (7.1)	1 (7.1)	0		0.011 (0.004–0.027)	0.724
≥ 6 months	62 (76.5)	8 (9.9)	5 (6.2)	6 (7.4)		0.015 (0.004–0.037)	
OS, months					0.458		
Median	85.5	61.7	74.9	89.3	0.847		
< 12 months	8 (100.0)	0	0	0		0.043 (0.003–0.071)	0.530
≥ 12 months	69 (75.0)	9 (9.8)	6 (6.5)	8 (8.7)		0.015 (0.004–0.034)	

Abbreviations: CR, complete response; IHC, immunohistochemistry; IQR, interquartile range; mRNA, messenger ribonucleic acid; NAC, neoadjuvant chemotherapy; OS, overall survival; PFS, progression‐free survival; PR, partial response; RT‐qPCR, quantitative reverse transcription polymerase chain reaction.

**FIGURE 4:**
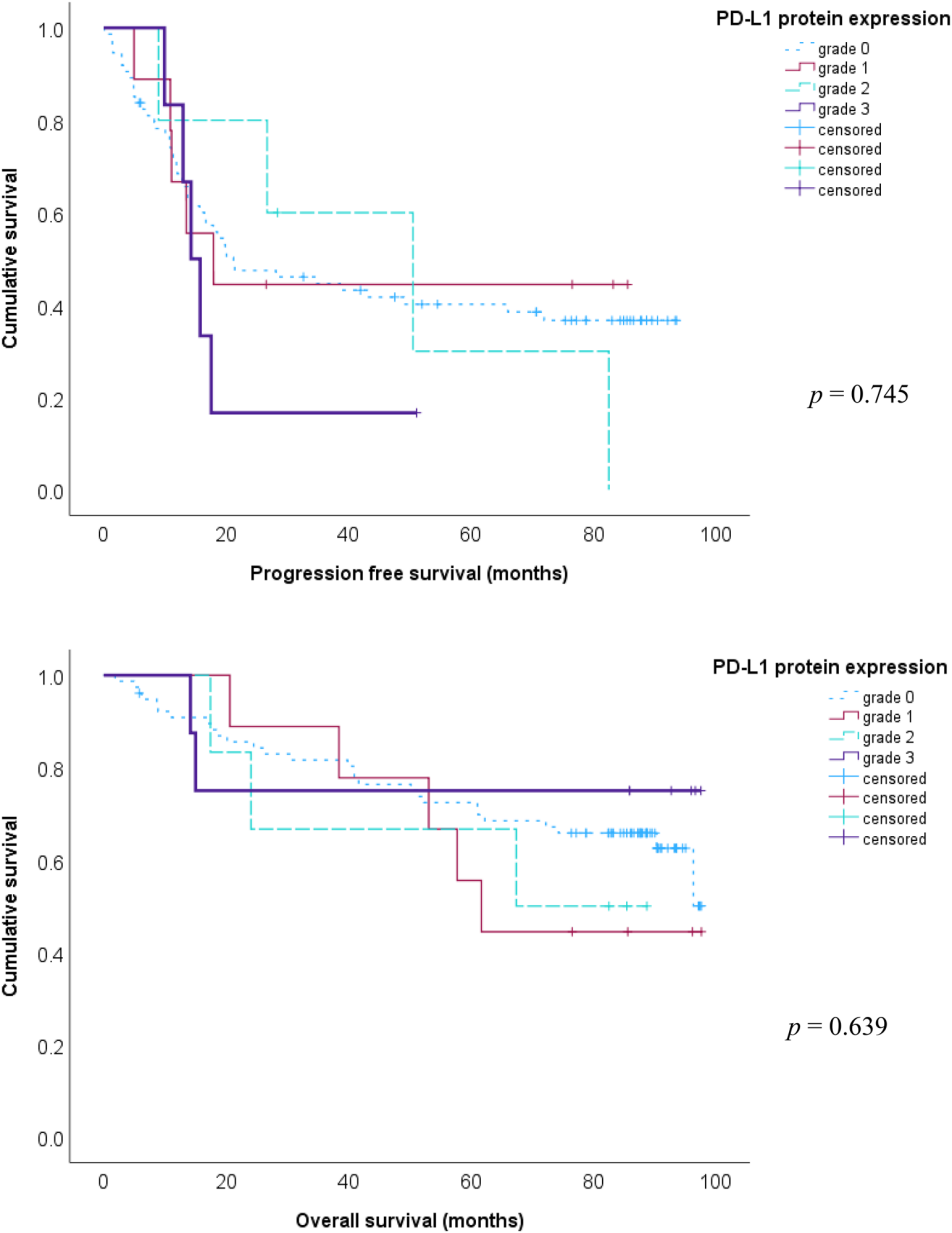
Progression‐free survival and overall survival curves of study patients classified by PD‐L1 protein expression.

Figure 4 presents the PFS and OS curves stratified by IHC staining grade. There were no significant differences in PD‐L1 expression grading for either PFS (*p* = 0.745) or OS (*p* = 0.639). Compared with IHC grade 0, the HRs for PFS were 0.907 (95% CI, 0.360–2.286), 1.085 (95% CI, 0.390–3.019), and 1.647 (95% CI, 0.648–4.185) for IHC grades 1, 2, and 3, respectively. Similarly, the HRs for OS were 1.515 (95% CI, 0.584–3.930), 1.529 (95% CI, 0.463–5.043), and 0.634 (95% CI, 0.150–2.675) for IHC grades 1, 2, and 3, respectively. Based on the grade of PD‐L1 expression, the mean PFS durations were 45.16 ± 4.63 months for grade 0, 44.53 ± 12.29 months for grade 1, 47.05 ± 14.89 months for grade 2, and 20.27 ± 5.72 months for grade 3. And the mean OS durations were 75.05 ± 3.81 months for grade 0, 69.12 ± 9.29 months for grade 1, 62.52 ± 12.47 months for grade 2, and 76.82 ± 12.70 months for grade 3. Exploratory analyses using dichotomized PD‐L1 expression cut‐offs likewise revealed no significant associations with clinicopathological characteristics or survival outcomes. Table 3 presents the multivariable Cox regression analysis of prognostic factors associated with PFS and OS. Advanced stage and high histologic grade were independently associated with shorter PFS, whereas advanced stage was the only independent prognostic factor associated with shorter OS.

**TABLE 3:** Multivariable Cox proportional hazards regression analysis of prognostic factors associated with progression‐free survival and overall survival.

Variables	Progression‐free survival		Overall survival	
	Univariate HR [95% CI]	Multivariate adjusted HR [95% CI]	Univariate HR [95% CI]	Multivariate adjusted HR [95% CI]
Stage				
Early, *n* = 51	Reference		Reference	
Advanced, *n* = 49	8.219 [4.296–15.725] *p* < 0.001	7.152 [3.730–13.713] *p* < 0.001	7.656 [3.184–18.407] *p* < 0.001	7.656 [3.184–18.407] *p* < 0.001
Histologic grade				
Low, *n* = 20	Reference		Reference	
High, *n* = 80	4.764 [1.720–13.195] *p* = 0.003	3.230 [1.153–9.051] *p* = 0.026	5.414 [1.303–22.498] *p* = 0.020	ns
Histology				
Serous, *n* = 42	Reference		Reference	
Non‐serous, *n* = 58	0.430 [0.255–0.725] *p* = 0.002	ns	0.678 [0.358–1.284] *p* = 0.233	NA
Surgical outcomes				
No residual, *n* = 52	Reference		Reference	
Residual ≤ 1 cm, *n* = 13	7.810 [3.611–16.895] *p* < 0.001	ns	7.467 [2.693–20.701] *p* < 0.001	ns
Residual > 1 cm, *n* = 35	4.829 [2.618–8.910] *p* < 0.001	ns	6.718 [2.868–15.734] *p* < 0.001	ns

Abbreviations: CI, confidence interval; HR, hazard ratio; NA, not applicable; ns, not statistically significant.

## 4 Discussion

Immune and inflammatory responses play crucial roles in oncogenic transformation, disease progression, and determine patients' outcomes. Various factors have been proposed and validated to predict cancer aggressiveness and triage treatment modalities. Tumor‐infiltrating lymphocytes (TILs) play a major role in anti‐tumor immunity and are a significant prognostic factor in EOC [13, 14]. The tumor microenvironment can escape host immunity via immune inhibitory checkpoint pathways; PD‐1/PD‐L1 interface [16]. The pathways potentially regulating PD‐L1 expression include adaptive immune resistance mediated by TILs, which have been shown to induce PD‐L1 expression. Additionally, tissue hypoxia promotes the production of cytokines that stimulate PD‐L1 expression [18, 23, 24, 25]. Regarding the PD‐1/PD‐L1 signaling pathway, monoclonal antibodies for cancer treatment have been studied. Immunohistochemistry staining is a mandatory pathology report to either determine the pathological subtype or differentiate the origin of primary cancers in sophisticated cases. For the most appropriate selection of patients, the use of immunohistochemistry staining as a predictive marker for response to specific immunotherapeutic agents has been investigated [23, 24, 26].

The current study showed that the proportion of PD‐L1 protein expression in cancer cells was 23%, which aligns with the findings of a previous EOC study. Webb et al. studied PD‐L1 protein staining (clone SP142) in tissue microarrays from tissue banking, which contained all histopathological subtypes of EOC. The proportion of positive cancer cells staining was 34.9% (171/490 patients), of which virtually all (169/171) patients had positive staining of the cancer cells and CD8+ [24]. This proportion was slightly higher than that found in the current study. In another study utilizing the SP263 clone in EOC, PD‐L1 expression was identified in stromal TILs, intraepithelial TILs, and tumor cells at rates of 16.9%, 10.5%, and 8.5%, respectively [27]. When looking at the effects of histopathology with PD‐L1‐positive cancer cells, a higher proportion of positive staining was found for the HGSC subtype (57.4%), and for histology grade 3 (38.8%), but without correlation with the cancer stage [24]. The proportion has been found to be as high as 60%–80% if one relies on immune cell staining or testing is restricted to the HGSC subtype [16, 18]. Using specific clone SP‐142 in ovarian HGSC tissue, PD‐L1 expression was observed in only 8% of tumor cells, whereas up to 74% of tumor‐associated macrophages expressed PD‐L1 [28]. A number of factors might be responsible for the variations in the proportion of PD‐L1 expression, such as the histopathological subtypes, histopathological gradings, cancer stages, time intervals from surgery to immunohistochemistry interpretation, tissue preserving techniques, specifics of the protein staining types, staining methods, and interpretation criteria.

Various methods for the analysis of gene expression have been used, including deoxyribonucleic acid (DNA) microarrays, DNA sequencing, mRNA expression, and proteomics analysis. The relationship between the protein and mRNA expression in cancers is not well understood [29]. The regulatory mechanisms controlling protein and post‐translational modifications in tumors are sophisticated. The current study demonstrated a moderate correlation coefficient between the PD‐L1 protein and PD‐L1 mRNA expression in EOC. This is similar to the relationship observed between PD‐L1 protein and mRNA expression in lung adenocarcinoma and pediatric solid tumors [30]. In contrast, a previous study in which the authors found a lack of correlation between PD‐L1 mRNA expression and PD‐L1 protein expression in ovarian HGSC cells [18]. These could occur for various reasons. Firstly, it may be due to the heterogeneity of the PD‐L1 expression in the tumor mass. Secondly, the mRNA expression was extracted from the whole tissue, including PD‐L1 from cancer and immune cells, but the immunohistochemistry was counted only in cancer cells. The last but not least, there might have been a selection bias in the tissue sampling.

In the current study, we did not demonstrate a predictive effect of PD‐L1 expression on survival outcomes. This may be due to differences in specific antibody clones and the relatively low PD‐L1 expression observed in the current study. Thus, the prognostic effects of PD‐L1 expression on solid tumors, including EOC, remain inconclusive. This is because the tumor microenvironment exhibits a broad complexity and impacts the biological significance of immune markers. This network and the importance of the different factors are not well understood. PD‐L1 IHC was performed by microarray cores, so the sampling bias might have influenced the low prevalence of positive PD‐L1 and may have limited the statistical power to detect survival differences.

Previously, the overexpression of PD‐L1 has been shown to predict poor OS in various solid tumors, including breast, renal, urothelial, and gastric cancers. Additionally, it is associated with poor PFS in melanoma, hepatocellular carcinoma, and renal carcinoma [31, 32]. In a meta‐analysis of PD‐L1 expression in EOC, they reported a PD‐L1 protein expression rate of 55.7% and observed a trend toward a worse prognosis [32]. For example, Hamanishi et al. studied their newly generated antibodies for PD‐L1 protein staining in CD8^+^ of 70 formalin‐fixed and paraffin‐embedded (FFPE) EOC tissues, comprising all histopathologic subtypes. The positive staining rate was 88.6%. They classified positive staining grade 0–1 as low expression, and staining grade 2–3 as high expression. The high‐expression group had significantly shorter PFS (5.92 ± 0.99 vs. 6.12 ± 0.72 years) and OS (6.48 ± 0.62 vs. 9.56 ± 0.82 years). Furthermore, the high expression group has been shown to be an independent poor prognostic factor of PFS and OS with adjusted OR 4.26, 95% CI 1.39, 12.94, *p* = 0.011 and adjusted OR 2.57, 95% CI 1.11, 5.93, *p* = 0.027, respectively [16].

In a subsequent study of PD‐L1 expression in EOC, the authors postulated that the PD‐L1 expression was an independent factor associated with a favorable disease‐specific survival in ovarian HGSC (HR 0.607, 95% CI 0.399, 0.925, *p* = 0.02) [24]. Moreover, coexistent cancer and CD8^+^ cells PD‐L1 expression was associated with a better prognosis (HR 0.219, 95% CI 0.067, 0.721) than CD8^+^ PD‐L1 expression alone [24]. In a study of FFPE specimens from ovarian HGSC patients, they performed two cores of tissue microarrays for each sample to studies of PD‐1/PD‐L1 expression in cancer cells and TILS. In addition, PD‐1/PD‐L1 mRNA expressions were evaluated by RT‐qPCR. They found the PD‐L1 protein expression in cancer cells of 86.4% (153/177 patients). The PD‐L1 protein expression in cancer cells was an independent prognostic factor for PFS, with a median PFS of 24.6 versus 14.3 months (HR 0.41, 95% CI 0.23, 0.71; *p* = 0.002). Additionally, 81.1% of study patients (142/175 patients) had a low PD‐L1 mRNA expression (mRNA ≤ 30.45). A high mRNA expression was an independent favorable prognostic for PFS (29.4 vs. 20.2 months; HR 0.41, 95% CI 0.23, 0.71; *p* = 0.001) [18]. Furthermore, the degree of PD‐L1 expression score was associated with favorable outcomes in response to immunotherapeutic agents. Moore et al. reported that a PD‐L1‐expressing immune cells score ≥ 5% predicted a positive response to atezolizumab in frontline treatment for stage III/IV EOC [33]. Additionally, a higher PD‐L1 CPS correlated with an increased response rate to pembrolizumab treatment in recurrent EOC, with response rates of 5.7% and 10% for CPS ≥ 1% and CPS ≥ 10%, respectively [34]. This supported a positive prognostic effect of cancer and CD8^+^ cells on PD‐L1 protein expression. It also corresponded with the presence of TIL and a better survival duration of patients with EOC, as proposed by previous studies [35, 36]. Similarly, the presence of the PD‐L1 protein confers a good prognosis in melanoma, non‐small cell lung carcinoma, colorectal carcinoma, and renal cell carcinoma [37, 38, 39].

This is the clinical prospective study design; there was also no time effect from the collection of the specimens or the paraffin retrieval process to the protein or mRNA evaluations. The surgical tissue was sampled by gynecologic oncologists at the most suspicious area for the protein and mRNA analyses. The PD‐L1 protein expression, which was evaluated based on cancer cells' expression, should represent cancer behaviors. We used triplicated RT‐PCR, which was normalized by two HKG to enhance the accuracy of the quantitative mRNA.

This study has several limitations. First, the relatively small sample size limited the statistical power to detect associations between PD‐L1 protein/mRNA expression and clinicopathological characteristics, surgical outcomes, and survival. Moreover, because no patients received immune checkpoint inhibitors, the predictive value of PD‐L1 expression for immunotherapy response could not be assessed. Therefore, the findings should be interpreted as biological and prognostic associations rather than evidence to guide treatment selection. Second, this was a single‐center study without internal or external validation cohorts, limiting the generalizability of the findings. In addition, PD‐L1 immunohistochemical scoring was performed by a single pathologist, and inter‐ and intraobserver reproducibility was not assessed, which may have introduced observer‐related variability. Validation in independent multicenter cohorts and external datasets, such as The Cancer Genome Atlas (TCGA), is warranted. Finally, PD‐L1 expression exhibits substantial spatial heterogeneity. Because immunohistochemical assessment was performed using tissue microarray cores, only a limited portion of each tumor was evaluated. Consequently, intratumoral and intertumoral heterogeneity could not be fully assessed, potentially leading to under‐ or overestimation of PD‐L1 expression and weakening its associations with clinicopathological features and clinical outcomes.

Future studies should incorporate multiple pathologists and formal concordance analyses to improve the reliability and reproducibility of PD‐L1 immunohistochemical scoring. Prospective, adequately powered studies are needed to evaluate the prognostic and predictive value of PD‐1/PD‐L1 expression for oncological outcomes, including treatment response, PFS, and OS, particularly in patients receiving immune checkpoint inhibitors. Standardization and validation of PD‐1/PD‐L1 assessment methods and clinically relevant cut‐off values are also essential to optimize patient selection for immunotherapy. In addition, the potential of PD‐1/PD‐L1 expression, alone or in combination with other molecular biomarkers, to predict platinum and taxane sensitivity in EOC warrants further investigation. Ultimately, integrating tumor microenvironment biomarkers into biologically informed predictive models may facilitate patient stratification and advance precision treatment strategies.

In conclusion, the expression of the PD‐L1 protein in epithelial ovarian/fallopian tube/primary peritoneal carcinoma in the current study was 23%, while the median expression of normalized mRNA was 0.015. There was no significant association of the PD‐L1 expression with the clinicopathological factors or survival outcomes. The moderate correlation between PD‐L1 protein and mRNA expression suggests partial concordance between transcriptomic and proteomic levels, reflecting complex post‐transcriptional regulation mechanisms. Future studies incorporating independent validation cohorts are required to confirm the clinical and biological relevance of PD‐L1 expression in EOC.

## Author Contributions

**Rachawalan Suriyasaengsri:** conceptualization, investigation, methodology, validation, visualization, data curation, resources, formal analysis, writing – review and editing. **Naravat Poungvarin:** supervision, data curation, resources, formal analysis, methodology, validation, visualization, writing – review and editing, conceptualization, investigation. **Irene Ruengkhachorn:** conceptualization, investigation, funding acquisition, visualization, validation, methodology, formal analysis, data curation, supervision, resources, writing – review and editing. **Pornprom Ittiamornlert:** conceptualization, investigation, methodology, validation, writing – review and editing. **Suchanan Hanamornroongruang:** conceptualization, investigation, methodology, validation, visualization, writing – review and editing, resources, data curation, supervision, formal analysis. **Achjima Tankul:** conceptualization, investigation, writing – original draft, methodology, validation, formal analysis, project administration, data curation, resources, software. **Sompop Kuljarusnont:** conceptualization, methodology, writing – review and editing, validation, investigation. **Saowalak Hunnangkul:** software, formal analysis, validation, writing – review and editing. **Wathirada Karnchanabanyong:** conceptualization, investigation, methodology, validation, writing – review and editing.

## Funding

This study was supported by a grant from the Siriraj Research Development Fund (R016033001). The funder had no role in study design, data collection and analyses, decision to publish, or preparation of the manuscript.

## Ethics Statement

This study was approved by the Siriraj Institutional Review Board (COA no. Si 694/2016 and COA no. Si 263/2024). The IRB‐approved consent form includes the objectives, potential risks and benefits of the study, and details the required tissue biopsies and the name of the principal investigator (A.T.) and corresponding investigator (I.R.) responsible for the protocol.

## Consent

The consent additionally specifies the patient's right to accept or refuse treatment. All participants gave written informed consent to participate in the study.

## Conflicts of Interest

The authors declare no conflicts of interest.

## Data Availability

Additional data and materials may be obtained from the corresponding author on reasonable request.
